# Editorial: The role of environmental stressors in neurocritical patient outcomes

**DOI:** 10.3389/fneur.2025.1614491

**Published:** 2025-09-30

**Authors:** Luis Rafael Moscote-Salazar, Tariq Janjua, Vishal K. Chavda

**Affiliations:** ^1^AV Healthcare Innovators, LLC, Madison, WI, United States; ^2^ANEUCLOSE, LLC, Eagan, MN, United States; ^3^Department of Medicine and Critical Care, Multispeciality, Trauma and ICCU Center, Sardar Hospital, Ahmedabad, Gujarat, India

**Keywords:** neurocritical care, critical care environment, extubation failure, neurological outcomes, environmental stressors

Neurocritical care occupies a unique nexus wherein demanding physiological management converges with the unpredictability of human environments. In this Research Topic, *The role of environmental stressors in neurocritical patient outcomes*, we sought to shed light on underexplored variables—often outside the traditional scope of intensive care protocol—that have the potential to profoundly impact outcomes in neurologically impaired patients.

The articles in this series reveal how environmental and systemic elements—from oxygenation, nutritional timing, tracheostomy care, and even pesticide exposure—can influence recovery, complications, and survival. Collectively, these studies challenge the conventional borders of neurocritical care and offer practical information for clinical practice.

Zhang et al. research article addressed a very basic procedural milestone in critical care: extubation. A large retrospective cohort examined risk factors for failed endotracheal extubation in neurocritical patients. By determining predictors like longer mechanical ventilation and reduced consciousness, the authors recommend more individualized readiness testing to prevent extubation failure and morbidity.

Complementing this, a synthesis of nursing and clinical care of tracheostomized patients with traumatic brain injury (Mao et al.) emphasized the multidisciplinary teamwork needed for airway care. It outlined how specific nursing interventions—viz., suction routines, stoma care, and communication support—can prevent complications and enhance comfort for patients.

Environmental toxicity was also highlighted in a dramatic case report of insecticide-induced leukoencephalomyelopathy (Li et al.). That hyperthermia was delayed in this lethal case highlights how exposure to agricultural chemicals is one of those rarely spoken but extremely important environmental threats to neurological health, especially in rural or underserved populations.

Meanwhile, the association of hematological markers with outcome was also explored in a study of thrombocytopenia in intracerebral hemorrhage patients (Feng et al.). This study found that platelet count trends can serve as both a biomarker and therapeutic target, again showing how systemic stress responses are mirrored in intracranial vulnerability.

On another level, a bibliometric analysis talked about the most cited articles on hypothermic brain protection (Hu et al.). The study mapped shifting scientific trends and knowledge clusters, offering a meta-perspective on how cooling therapies have shaped neurocritical practices over the past decades.

Nutrition timing constituted another modifiable stressor in a study using a MIMIC-IV database of stroke patients (Wang X. et al.). The authors associated early enteral feeding with reduced 28-day mortality, highlighting the necessity of nutritional planning within the acute period of neurologic recuperation.

This theme of time-dependent care was corroborated in a retrospective single-center study examining the combined effect of mechanical thrombectomy and prolonged mild hypothermia in acute middle cerebral artery occlusion (Wang A. et al.). The findings suggest synergistic effects in the application of both mechanical and environmental modes of treatment for ischemic stroke.

Circumambient oxygenation was also a powerful variable in a MIMIC-IV analysis of non-traumatic subarachnoid hemorrhage (Liu et al.). Transcutaneous oxygen saturation levels during the first 24 h predicted in-hospital mortality, offering an easily accessed but powerful prognostic marker that may be responsive to early environmental and respiratory support interventions.

At a systems modeling level, a predictive model was developed to calculate the likelihood of return of spontaneous circulation and favorable neurological outcomes following in-hospital cardiac arrest (Li and Xing). By consolidating clinical and demographic variables, this tool demonstrates how data-driven frameworks can streamline bedside decision-making along with potentially informing environmental priorities following resuscitation.

Lastly, a network meta-analysis compared screening tools for dysphagia in stroke patients (Jiang et al.). The results emphasize how early and personalized screening prevents aspiration, reduces ICU-acquired infections, and improves outcomes—a testament to how procedural timing and environment can be a therapeutic axis.

Collectively, this Research Topic shines a light on the insidious and unrecognized environmental influences on neurocritically ill patient outcomes. From air quality and sedation to the timing of nutrition and human presence, every modifiable aspect of care can either optimize recovery or impart risk.

We thank all contributors, peer reviewers, and readers for making this Research Topic a robust and thought-provoking exploration of an emerging frontier in neurological care. We hope that these articles will inspire clinicians and researchers alike to further explore and integrate environmental perspectives in neurocritical care protocols.

